# Identification of fungal natural products with potent inhibition in *Toxoplasma gondii*

**DOI:** 10.1128/spectrum.04142-23

**Published:** 2024-02-29

**Authors:** Tiantian Jiang, Karla P. Godinez-Macias, Jennifer E. Collins, Jin Woo Lee, Karen L. Wendt, Krypton Carolino, Debopam Chakrabarti, Robert H. Cichewicz, Elizabeth A. Winzeler

**Affiliations:** 1 Department of Pediatrics, School of Medicine, University of California, San Diego, La Jolla, California, USA; 2 Division of Molecular Microbiology, Burnett School of Biomedical Sciences, University of Central Florida, Orlando, Florida, USA; 3 College of Pharmacy, Duksung Women’s University, Seoul, Republic of Korea; 4 Natural Products Discovery Group, Department of Chemistry and Biochemistry, University of Oklahoma, Norman, Oklahoma, USA; Hubei University of Medicine, Shiyan, Hubei, China

**Keywords:** *Toxoplasma gondii*, fungal natural products, scaffold discovery, xanthoquinodin, peptaibol, heptelidic acid, fumagillin

## Abstract

In an effort to identify novel compounds with potent inhibition against *Toxoplasma gondii,* a phenotypic screen was performed utilizing a library of 683 pure compounds derived primarily from terrestrial and marine fungi. An initial screen with a fixed concentration of 5 µM yielded 91 hits with inhibition comparable to an equal concentration of artemisinin. These compounds were then triaged based on known biological and chemical concerns and liabilities. From these, 49 prioritized compounds were tested in a dose response format with *T. gondii* and human foreskin fibroblasts (HFFs) for cytotoxicity. Ten compounds were identified with an IC_50_ less than 150 nM and a selectivity index (SI) greater than 100. An additional eight compounds demonstrated submicromolar IC_50_ and SI values equal to or greater than 35. While the majority of these scaffolds have been previously implicated against apicomplexan parasites, their activities in *T. gondii* were largely unknown. Herein, we report the *T. gondii* activity of these compounds with chemotypes including xanthoquinodins, peptaibols, heptelidic acid analogs, and fumagillin analogs, with multiple compounds demonstrating exceptional potency in *T. gondii* and limited toxicity to HFFs at the highest concentrations tested.

**IMPORTANCE:**

Current therapeutics for treating toxoplasmosis remain insufficient, demonstrating high cytotoxicity, poor bioavailability, limited efficacy, and drug resistance. Additional research is needed to develop novel compounds with high efficacy and low cytotoxicity. The success of artemisinin and other natural products in treating malaria highlights the potential of natural products as anti-protozoan therapeutics. However, the exploration of natural products in *T. gondii* drug discovery has been less comprehensive, leaving untapped potential. By leveraging the resources available for the malaria drug discovery campaign, we conducted a phenotypic screen utilizing a set of natural products previously screened against *Plasmodium falciparum*. Our study revealed 18 compounds with high potency and low cytotoxicity in *T. gondii*, including four novel scaffolds with no previously reported activity in *T. gondii*. These new scaffolds may serve as starting points for the development of toxoplasmosis therapeutics but could also serve as tool compounds for target identification studies using chemogenomic approach.

## INTRODUCTION

*Toxoplasma gondii* (*T. gondii*) can infect virtually all warm-blooded birds and mammals, including humans. It is estimated that one-third of the human population is chronically infected with *T. gondii* (1). The acute stage of *T. gondii* infection is characterized by the rapid replication of tachyzoites. To evade the host immune system*,* these tachyzoites then transform to become semi-dormant bradyzoites, which marks the start of the chronic stage of toxoplasmosis. Infection in immunocompromised individuals, newborns, and developing fetuses is often fatal. However, to date, no vaccine or fully efficacious drugs are available for prophylactic and therapeutic treatment. A combination of pyrimethamine and sulfadiazine is the current gold standard therapy for *T. gondii*. These two drugs act synergistically to disrupt DNA synthesis by interfering with folate production. During therapy, folinic acid (leucovorin) can be supplemented to restore the host's folate levels without impacting the parasite due to a lack of membrane transport mechanisms in *T. gondii* for exogenous folate. Unfortunately, sulfadiazine can induce side effects such as allergies and bone marrow suppression (2). As a result, sulfadiazine is sometimes replaced with a protein synthesis inhibitor such as azithromycin, or an electron transport inhibitor such as atovaquone (3). Cerebral and ocular toxoplasmosis are the most challenging forms of infection to treat, as drugs must cross the blood brain barrier to reach the target sites at an effective concentration. For cerebral toxoplasmosis, in addition to pyrimethamine and sulfadiazine, trimethoprim combined with sulfamethoxazole (co-trimoxazole) or atovaquone can be used. Clindamycin or pyrimethamine-sulfadiazine in combination with corticosteroids are also given to treat ocular toxoplasmosis. However, resistance and treatment failures are well documented with pyrimethamine and sulfadiazine (2). Without long-term treatment, 80% of the cases will relapse and around 40% of the patients may discontinue treatment due to side effects (2). Drugs are also needed for treating bradyzoites, the chronic stage of *T. gondii*. Together, these challenges highlight the need to develop novel and efficacious therapeutics for the treatment of toxoplasmosis.

Natural products have historically been a major source of therapeutics for a variety of parasitic diseases (4). In addition to containing unexplored potential, natural products offer greater diversity, structural complexity, and molecular rigidity over synthetic molecules (4). Some of the most successful natural products to date include the antimalarials artemisinin and quinine. Additional screening of natural product libraries could provide exciting and unique starting scaffolds with the possibility for further optimization. To that end, we performed a phenotypic screen of a natural product library containing 683 fungal derived compounds against *T. gondii* tachyzoites in order to identify potent and selective inhibitors. This library had previously been cross-screened against the related apicomplexan parasite, *Plasmodium falciparum* (Dd2) by utilizing a SYBR Green I fluorescence-based assay (5). This work identified 78 unique compounds with greater than or equal to 50% inhibition at 1 µM, of which 57 demonstrated an EC_50_ less than 1 µM. Additionally, the cytotoxicity of these compounds was determined in HepG2 cells, and scaffolds with high potency and low cytotoxicity were selected for further study (5).

## RESULTS

**Fig 1:**
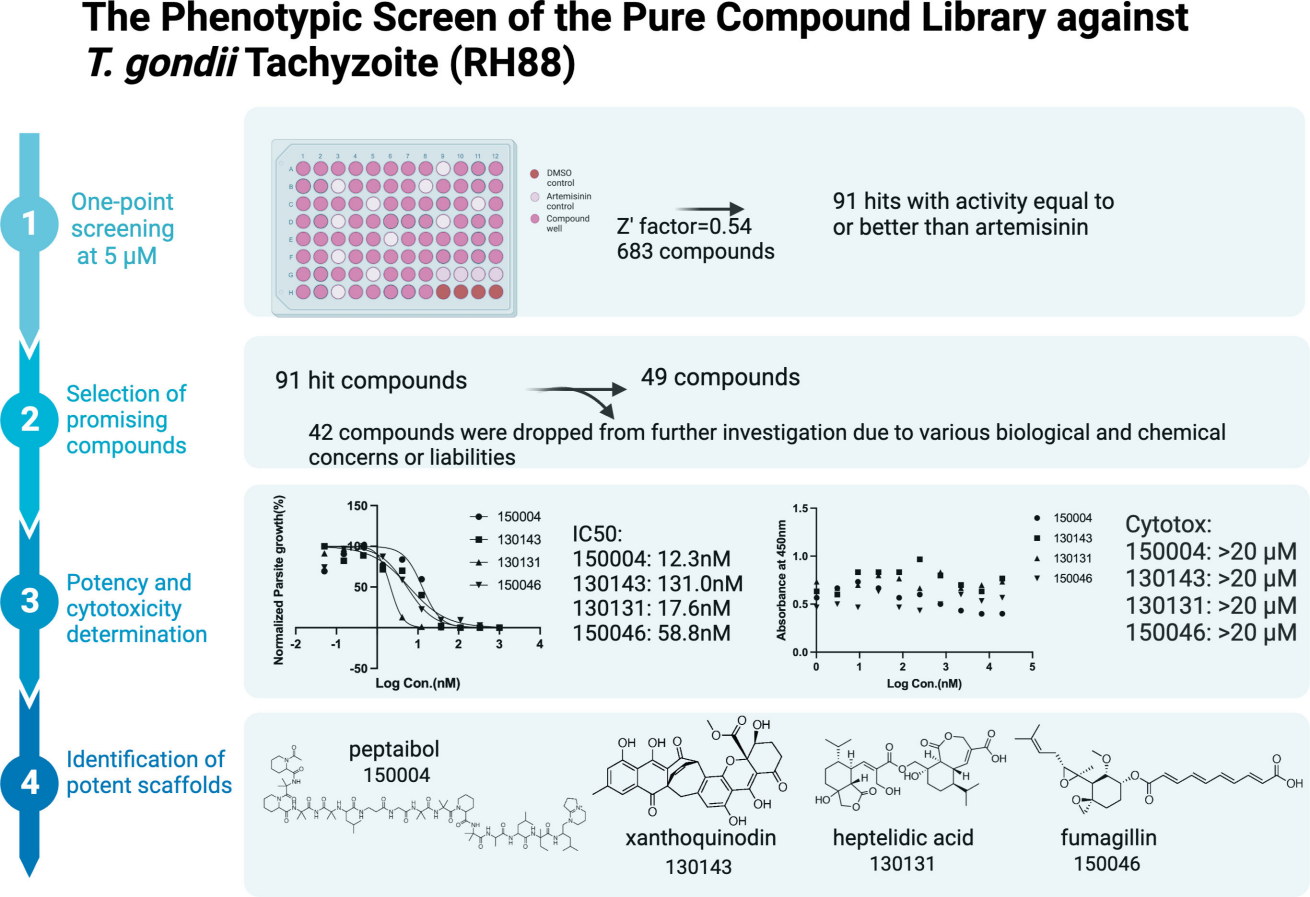
The workflow of phenotypic screen of the pure compound library against *Toxoplasma gondii* tachyzoites (RH88). One point screening at 5 µM yields 91 hits that have activity similar or better than artemisinin at the same concentration. Forty-nine compounds were selected for potency determination and cytotoxicity measurement. Four novel scaffolds were identified in addition to the reported cyclic tetrapeptide analogs. This figure was created with BioRender.com.

This screen of 683 pure natural products at 5 µM yielded 91 initial hits with at least 94% inhibition, equal to or greater than the inhibition of the same concentration of artemisinin. Pyrimethamine or sulfadiazine was not used as positive controls due to the resistance of the strain to these drugs. This strain of RH88 was genetically engineered to express luciferase and had an insertion of DHFR as selection marker (6). The average Z′ score across all plates was 0.54 (Fig. 1).

Upon review of the initial set of hit molecules, 42 compounds were triaged out due to various biological and chemical concerns such as poor chemical stability, known human toxicity, compound availability, and target promiscuity. This resulted in a curated subset of 49 fungal metabolites with promising chemical properties and biological activities that warranted further investigation. These compounds were then subjected to IC_50_ determination and cytotoxicity screening. To test the IC_50_ assay, we used three positive controls that have IC_50_ data published in *T. gondii*, namely, KAE609, artemisinin, and methylene blue. The IC_50_ values of these compounds (Table 1) were found to be comparable to published values (7). With the method validated, we generated the IC_50_ data of the 49 compounds as shown in Table 1.

**TABLE 1:** *T. gondii* and HFF activities of the 49 prioritized natural products

Compound group	Name	NPDG ID	*Tg*RH88 IC_50_ (nM) (mean ± SEM)	HFF IC_50_ (nM) (mean ± SEM)	SI	*P. falciparum* hit (Y/N)	*Pf*Dd2 IC_50_ (nM)^*a*^	Potential target in *P. falciparum*
Peptaibols	Efrapeptin F	150003	55.5 ± 11.8	>20,000	>360	Y	399^*b*^	-
	Efrapeptin G	150004	12.3 ± 2.3	>20,000	>1,626	Y	460^*b*^	-
Verticillin Analogs	-	130039	168.6 ± 156.4	1,140.3 ± 104.9	7	Y	732	Histone methyltransferase^*c*^
	-	130044	86.6 ± 25.2	156.4 ± 2.6	2	N	n/a	
	Verticillin D	130016	36.2 ± 1.8	943.2 ± 6.4	26	N	n/a	
	Verticillin	130038	79.0 ± 29.3	82.4 ± 41.8	1	Y	501	
	Leptosin A	130037	12.1 ± 8.1	538.8 ± 77	45	Y	730	
	-	170042	65.6 ± 6.9	377.9 ± 26.5	6	Y	>4,600	
Heptelidic acid analogs	-	130131	17.6 ± 10.3	>20,000	>1,136	Y	281	Possible GAPDH inhibitor
	Chlorohydrin	130129	1,116.4 ± 34.9	>20,000	>18	Y	337	
	-	130130	>6,360.5	>20,000	-	Y	438	
Cyclic Tetrapeptides	Apicidin B	120250	63.2 ± 18.9	>40,000	>633	Y	352^*d*^	HDAC inhibitor^*d*^
	Apicidin C	120251	25.9 ± 10.2	>20,000	>772	Y	236	
	1-Alaninechlamydocin	130134	28.7 ± 11.0	>20,000	>697	Y	100^*e*^	
	Apicidin analog	120249	>5,899.5	>40,000	-	N	n/a	
Xanthoquinodin Analogs	Xanthoquinodin A1	130144	111.7 ± 74.0	12,886.4 ± 1,912	115	Y	250	-
	Xanthoquinodin A2	130147	56.4 ± 12.8	12,308.7 ± 313.8	218	Y	680	-
	Xanthoquinodin B2	130143	131.0 ± 36.5	>20,000	>153	Y	1,010	-
Pyridochromenes	PF-1140	120159	3,292.8 ± 107.0	>20,000	>6	N	n/a	-
	-	160005	1,508.0 ± 444.1	>20,000	>13	N	n/a	-
	-	160006	>5,607.0	>20,000	-	N	n/a	-
Calphostins	-	170069	3,468.9 ± 28.1	>40,000	>12	N	n/a	-
	-	170070	>6,678.1	>40,000	-	N	n/a	-
	-	170067	>5,447.9	>20,000	-	Y	390	-
	Calphostin C	170068	1,143.5 ± 261.6	>20,000	>17	Y	410	-
Ungrouped Scaffolds	RES-1149–2	150125	249.3 ± 74.1	>60,000	>241	Y	3,490	-
	-	150023	503.7 ± 15.5	>20,000	>40	N	n/a	-
	-	150029	585.2 ± 14.6	28,547.4 ± 2,365.2	49	N	n/a	-
	-	170030	403.6 ± 90.9	36,919.6 ± 706.6	91	N	n/a	-
	Polluxochrin	150052	801.0 ± 162.8	>60,000	>75	N	n/a	-
	Alpha-cyclopiazonic acid	170027	1,126.5 ± 496.0	>40,000	>36	N	n/a	-
	-	150068	938.6 ± 11.9	39,791.0 ± 939.5	42	N	n/a	-
	Fumagillin	150046	58.8 ± 7.3	>20,000	>340	Y	1,310	PfMetAP2^*e*^
	-	120157	>18,394.7	>20,000	-	N	n/a	-
	-	120152	4,641.5 ± 861.5	>20,000	>4	N	n/a	-
	Secopenitrem D	120253	14,108.7 ± 623.8	>20,000	>1	N	n/a	-
	Secalonic acid D	130002	334.4 ± 102.8	3,734.2 ± 1,359.4	11	Y	1,300	-
	-	130035	>5,725.4	>20,000	-	N	n/a	-
	Pyridoxatin	130034	>8,559.1	>20,000	-	N	n/a	-
	-	130073	>6,449.3	16,203.2 ± 4,040	<3	N	n/a	-
	Dinapinone A	150020	3,170.2 ± 28.55	6,674.8 ± 1,871.8	2	N	n/a	-
	-	150035	554.6 ± 4.5	1,748.2 ± 246.6	3	Y	980	-
	Brefeldin A	160010	243.4 ± 78.4	6,429.1 ± 94	26	N	n/a	-
	-	170003	>5,604.9	13,006.8 ± 864	<2	Y	1,470	-
	Aspochalasin D	170081	1,273.3 ± 454.5	30,270.3 ± 3,385.1	24	N	n/a	-
	-	170096	3,808.6 ± 260.7	>40,000	>11	N	n/a	-
	-	170085	425.5 ± 68.1	15,058.7 ± 3,532.3	35	N	n/a	-
	Chetomin	170066	62.2 ± 3.75	368.9 ± 72.6	6	N	n/a	-
	-	170078	587.1 ± 4.9	1,128.8 ± 428.0	2	Y	170	-
Positive controls	KAE609	-	439.5 ± 34.8	>20,000	>46	-	-	ATP4^*f*^
	Methylene blue	-	258.0 ± 0.95	>20,000	>78	-	-	Uncertain^*f*^
	Artemisinin	-	702.3 ± 24.7	10,482.3 ± 536.5	15	-	-	Peroxide-mediated oxidative damage (Kelch 13)^*f*^

^*a*^ Values represent the mean of duplicate or triplicate experiments.

^*b*^ Lee et al. (8).

^*c*^ Huber et al. (9).

^*d*^ Collins et al. (10).

^*e*^ Chen et al. (11).

^*f*^ Radke et al. (7).

**Fig 2:**
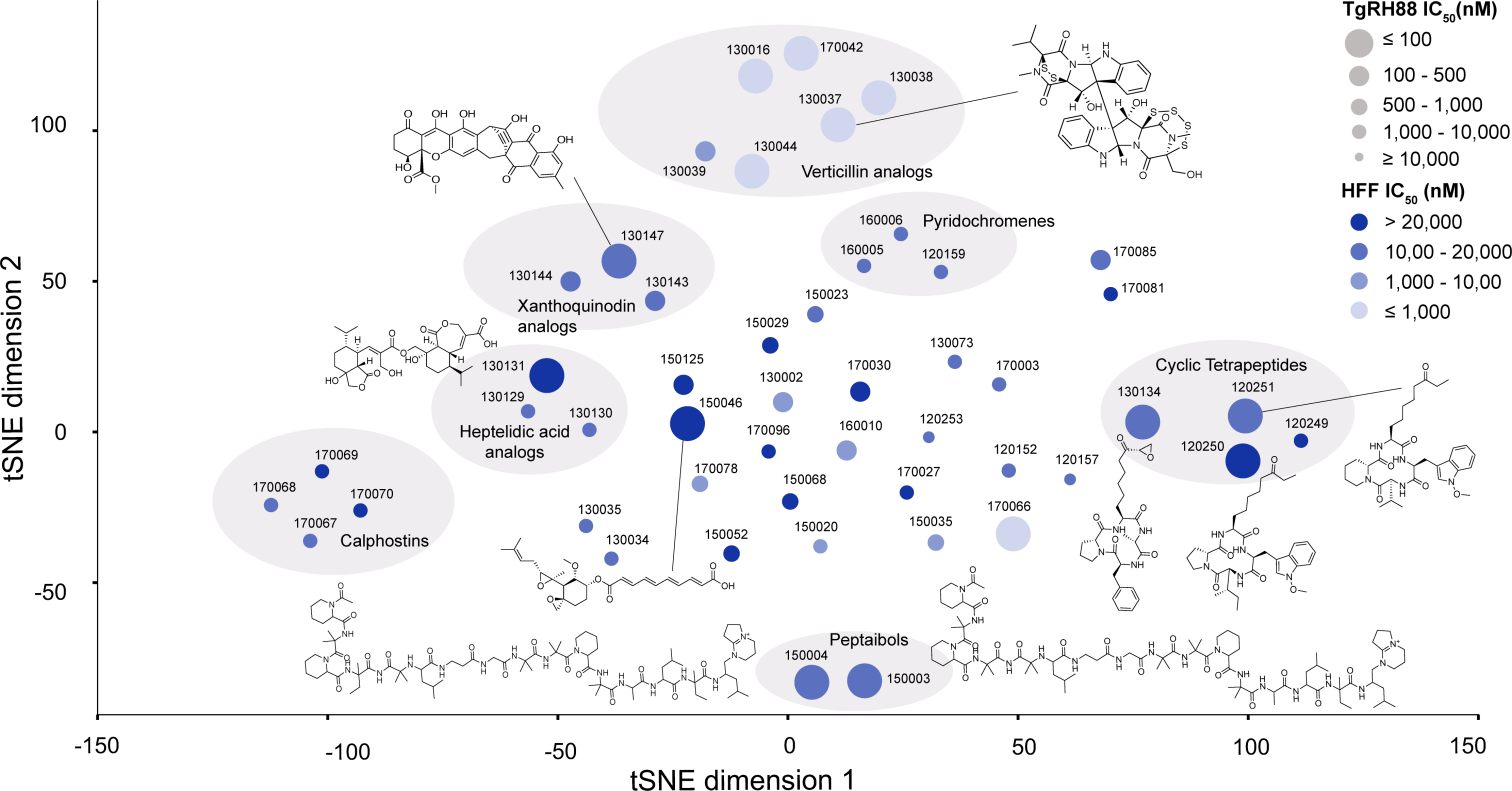
Clustering analysis of selected 49 hit compounds. SMILES for 49 hit compounds were converted using Morgan fingerprint with radius 4 and 2048 bits as determined by RDKIT. Clusters were assigned using Tanimoto similarity threshold of 80%, indicated by light gray circles. T-distributed stochastic neighbor embedding (t-SNE) was used to visualize fingerprint similarities in a scatter plot. Each dot represents a unique hit compound and scaffold categories are indicated when known. IC_50_ (nM) was represented by dot size with bigger dot size representing lower IC_50_ and vice versa. The node's color intensity indicates the cytotoxicity IC_50_ (nM) with the darker the color, the lower the cytotoxicity. Probability values were calculated using the hypergeometric mean function. Clusters all had enrichment values greater than expected by chance. Structures for the notably efficacious compounds were shown.

**Fig 3:**
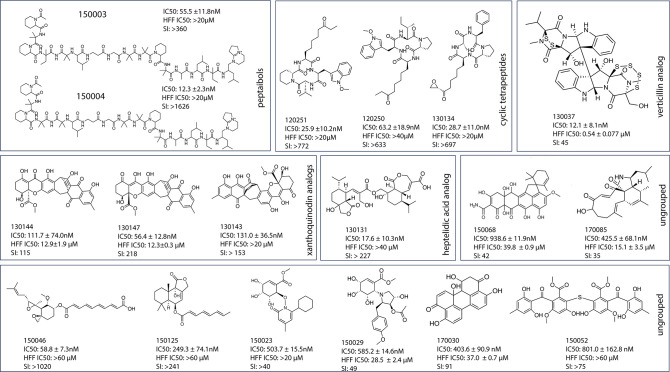
The chemical structures and IC_50_ values of the 18 potent compounds.

Chemoinformatic analysis on the 49 compounds identified seven clusters with enrichment at rates greater than those expected by chance (*P* = 5.11 **×** 10^−4^ – 1.62 **×** 10^−2^) (Fig. 2). Among these clusters, four scaffold groups stood out as containing compounds with high potency and low cytotoxicity. These included the peptaibol, xanthoquinodin, heptelidic acid, and cyclic tetrapeptide scaffold groups. In addition, fumagillin stood out as a single ungrouped scaffold. For further analysis, we focused our efforts on 18 compounds with submicromolar activity and favorable selectivity, of which 10 demonstrated an IC_50_ less than 150 nM (Table 1; Fig. 3).

## DISCUSSION

As previously mentioned, this compound library had also been tested against the *P. falciparum* parasite line Dd2 (5). Many of the inhibitors demonstrated dual activity in both protozoan parasites (Table 1), suggesting similar possible drug targets in the two closely related species. Overall, higher potency and selectivity was observed in *T. gondii* compared to *P. falciparum*. The most significant difference was noted with the peptaibol compounds. For example, efrapeptin F (150003) and efrapeptin G (150004) were found to be 7 and 37 times more potent in *T. gondii*, respectively (Table 1). These two peptaibols also showed no toxicity to human foreskin fibroblasts (HFFs) at the highest concentrations tested (20 µM). Peptaibols are membrane-active polypeptides characterized by the presence of a non-ribosomally synthesized amino acid, α-aminoisobutyric acid, and a C-terminal hydroxylated amino acid (Fig. 3). To date, no studies have reported the inhibitory activity of peptaibols in *T. gondii*, although they have been implicated against the malaria parasites (8). These two 16-amino-acid peptaibols were procured from *Tolypocladium sp.* and were originally shown to have insecticidal and antimicrobial activity (12). Whether fungus-derived peptaibols from other compound groups have activity in *T. gondii* is still unknown. These findings highlight the promise of this unique compound class for peptaibol-driven anti-*T*. *gondii* drug discovery.

The second noteworthy scaffold group was the xanthoquinodin analogs. These compounds demonstrated broad spectrum activity against numerous human pathogens including *Mycoplasma genitalium*, *P. falciparum, Cryptosporidium parvum*, *Trichomonas vaginalis*, and others (13). As seen with the previous antimalarial screening, xanthoquinodin A1 (130144) and A2 (130147) demonstrated the greatest activity with *T. gondii* IC_50_ values of 111.7 and 56.4 nM, respectively. Dissimilar to the results in *Plasmodium*, xanthoquinodin A2 showed slightly higher potency and selectivity than the A1 analog. Additionally, both xanthoquinodin A1 and A2 were found to be 2 and 12 times more potent in *T. gondii* as compared to *P. falciparum*, respectively. This, again, highlights the potential of these compounds for use in *T. gondii* drug discovery.

A third scaffold group identified was the cyclic tetrapeptides, including 1-alaninechlamydocin (130134) that showed a *T. gondii* IC_50_ of 28.7 nM and a SI of greater than 697. Other members of this group included apicidin B (120250) and C (120251) that demonstrated IC_50_ values of 63.2 and 25.9 nM against *T. gondii*, respectively. Cyclic tetrapeptides such as apicidin have been shown to act as histone deacetylase (HDAC) inhibitors in *Plasmodium* (10). These compounds in particular are suspected to inhibit the plasmodial class I HDAC (PfHDAC1) (13), causing rapid hyperacetylation of histone H4 *in vitro* using *P. falciparum*. The anti-*T*. *gondii* activity of these apicidin analogs was also previously reported (14). One analog (FR235222) demonstrated success in inhibiting not only tachyzoites but also bradyzoites in cysts (15). Additional studies later identified the drug target of FR235222 as *T. gondii* HDAC3 (TgHDAC3) (16). While high cytotoxicity of these compounds was indicated in other cell lines (e.g., HepG2), we observed low cytotoxicity in HFFs.

Several verticillin analogs were also identified with *T. gondii* potency. Verticillins are a group of compounds made up of fungal 1,4-bridged epiplythiodioxopiperazines (ETPs). Their structures consist of two ETP monomers connected through a C-C bond joining two quaternary carbons. Although the members of this group had low IC_50_ values against *T. gondii*, they were highly cytotoxic, and therefore possessed poor selectivity. Leptosin A is the exception with an IC_50_ of 12.1 nM and SI of 45. Verticillins have demonstrated activity in a wide range of microbes including bacteria and nematodes and were found to be cytotoxic to many cancer cell lines including HeLa cells (17). The activity of this group is attributed to their sulfide bridge that targets proteins via a reaction with thiol groups, leading to toxicity through the production of reactive oxygen species (17). In previous work, verticillin A has shown to target histone methyltransferases, which are epigenetic regulators like HDACs (9).

Other notable hits include the heptelidic acid analog 130131, which demonstrated high potency against *T. gondii* (IC_50_ of 17.6 nM) and good selectivity (SI greater than 1136). In other organisms, heptelidic acid was shown to inhibit GAPDH (glyceraldehyde 3-phosphate dehydrogenase) through the formation of a covalent bond between the thiol group of the catalytic cysteine and the epoxide ring of heptelidic acid (18). Heptelidic acid has also been found to have heme-dependent antimalarial activity and good murine bioavailability *in vivo* (19). There is limited research regarding the activity of these heptelidic acid analogs in *T. gondii,* although a large-scale screening at 100 µM found inhibition with the analog chlorohydrin, while the analog hydroheptelidic acid showed no activity (20).

Finally, we identified the fumagillin analog 150046 with an IC_50_ of 58.8 nM against *T. gondii*. Zhang et al. (21) had previously shown that fumagillin and its synthetic analog TNP-470 were active in malaria parasites (21). Fumagillin is known to irreversibly inhibit the human type 2 methionine aminopeptidase (MetAP2) and was explored as an anticancer agent due to its inhibition of angiogenesis. Additionally, in a pull-down assay, fumagillin was found to bind to PfMetAP2, the human ortholog of MetAP2 (11). A clinical trial of TNP-470 as a cancer therapeutic was halted due to its neurotoxicity and short half-life *in vivo* (22). To reduce the neurotoxicity and pharmacological activities of fumagillin-based analogs, researchers created fumarranol that was found to have activity in *P. falciparum* Dd2 and 3D7 *in vitro* and to bind *in vivo* to PfMetAP2 (11). Beloranib, an analog of fumagillin, was advanced to phase II clinical trials due to its effect on weight loss (23). However, its phase III clinical trial was halted due to patient deaths. Given the high potency in *T. gondii* of fumagillin analog 150046, and its lack of toxicity at the highest concentration tested (60 µM), this compound group may warrant further study and development.

### Conclusion

In conclusion, a screening of natural products primarily derived from fungi identified potent anti-*T*. *gondii* scaffolds, many of which show dual activity in *P. falciparum*. Overall, these inhibitors were more active in *T. gondii*, highlighting the potential advantages of reutilizing antimalarial screening libraries against other protozoan parasites. Additionally, having the established activity in a closely related species allows for the prioritization of compounds based on known activity and the potential of shared targets. While some of the inhibitors identified were previously known, many had not yet been implicated in *T. gondii*. This current discovery of potent inhibitors could spearhead *T. gondii* drug discovery featuring these scaffolds, opening up promising new research avenues in the search for anti-*T*. *gondii* therapeutics.

## MATERIALS AND METHODS

For the initial fixed concentration screening and IC_50_ determination in *T. gondii*, we utilized a luciferase expressing RH88 strain to quantify the number of live, actively replicating parasites. To construct this strain, the Fluc and DHFR selection markers had been integrated into the UPRT locus through CRISPR/Cas9, rendering the strain pyrimethamine-resistant (6). Screening was performed using a pure compound library provided by the University of Oklahoma Natural Products Discovery Group, containing 683 compounds derived primarily from fungal secondary metabolites. For purposes of testing, HFFs were grown in 96-well microtiter plates in preparation. The media we used to grow cells was referred to a D10 media that was composed of Dulbecco's Modified Eagle Medium (Thermo Fisher; Cat # 11995073), 10% Fetal Bovine Serum (Sigma-Aldrich; Cat # 12306C), 1 **×** non-essential amino acids (100 **×**; Thermo Fisher; Cat # 11140050), HEPES buffer (1M; Fisher Scientific; Cat # SH3023701) at 0.4M, and 10 mg/mL of gentamicin (Fisher Scientific; Cat # 15710072). Compounds were then diluted to 10 µM in 100 µL of fresh D10 media. Each plate utilized artemisinin and DMSO vehicle controls. To each well, 2,500 parasites per 100 µL of D10 media were added. Three technical replicates were then performed. After 48 hours, 160 µL of the D10 media was removed, and plates were subjected to freezing then thawing. Following this, 10 µL of the passive lysis buffer (Promega; Cat# E1910) was added and incubated at room temperature for 15 min. Finally, 100 µL of luciferase assay reagent (Promega; Cat# E1501) was added to each well, and luminescence was measured using a PHERAstar FSX plate reader (BMG Labtech, Germany). Parasite inhibition was calculated based on the controls, with hits defined as those compounds with a mean potency equal to or greater than artemisinin (94% inhibition) in all three wells.

For IC_50_ and cytotoxicity determination, we used 384 plates with confluent HFF cells. Compounds were serially diluted 3-fold with a starting concentration of 20 µM. Samples were further diluted by adding an equal volume of media containing 700 parasites. DMSO and 40 µL of KAE609 were used as negative and positive controls, respectively. Assays were performed with a total of three technical replicates and two biological replicates. After incubation for 48 hours, 50 µL of media was removed, and plates were subjected to freezing and thawing. Following this, 10 µL of passive lysis buffer was added and plates were incubated for 15 min at room temperature. Finally, 30 µL of luciferase reagent was added, and the luminescence was measured immediately using a PHERAstar FSX. In parallel, cytotoxicity dose response was performed in the same fashion without the addition of parasites. Cell counting kit-8 reagent (GlpBio; Cat # GK10001) was added at a 1:10 dilution and incubated at 37°C, 5% CO_2_ for 1 hour. The absorbance was then measured at 450 nm using a PHERAstar FSX. The data management system CDD vault was used for curve fitting and IC_50_ determination. In the same fashion, the IC_50_ values of three positive controls (KAE609, artemisinin, and methylene blue) were measured (Table 1) and found to be comparable to published values (7).

To investigate inhibitory patterns within the structural diversity of these 49 hits, a chemoinformatic analysis was performed. The SMILES of each compound were converted to Morgan fingerprints using RDKIT (RDKit: Open-source cheminformatics. https://www.rdkit.org). Compounds were clustered together if they shared at least 80% Tanimoto similarity. Clusters and singletons were spatially arranged using fingerprint distances and visualized with Matplotlib (v3.7).
